# Comparative Chloroplast Genomes of *Camellia* Species

**DOI:** 10.1371/journal.pone.0073053

**Published:** 2013-08-23

**Authors:** Jun-Bo Yang, Shi-Xiong Yang, Hong-Tao Li, Jing Yang, De-Zhu Li

**Affiliations:** 1 Germplasm Bank of Wild Species, Kunming Institute of Botany, Chinese Academy of Sciences, Kunming, Yunnan, China; 2 Key Laboratory of Biodiversity and Biogeography, Kunming Institute of Botany, Chinese Academy of Sciences, Kunming, Yunnan, China; Cankiri Karatekin University, Turkey

## Abstract

**Background:**

*Camellia* , comprising more than 200 species, is a valuable economic commodity due to its enormously popular commercial products: tea leaves, flowers, and high-quality edible oils. It is the largest and most important genus in the family Theaceae. However, phylogenetic resolution of the species has proven to be difficult. Consequently, the interspecies relationships of the genus *Camellia* are still hotly debated. Phylogenomics is an attractive avenue that can be used to reconstruct the tree of life, especially at low taxonomic levels.

**Methodology/Principal Findings:**

Seven complete chloroplast (cp) genomes were sequenced from six species representing different subdivisions of the genus *Camellia* using Illumina sequencing technology. Four junctions between the single-copy segments and the inverted repeats were confirmed and genome assemblies were validated by PCR-based product sequencing using 123 pairs of primers covering preliminary cp genome assemblies. The length of the *Camellia* cp genome was found to be about 157kb, which contained 123 unique genes and 23 were duplicated in the IR regions. We determined that the complete *Camellia* cp genome was relatively well conserved, but contained enough genetic differences to provide useful phylogenetic information. Phylogenetic relationships were analyzed using seven complete cp genomes of six *Camellia* species. We also identified rapidly evolving regions of the cp genome that have the potential to be used for further species identification and phylogenetic resolution.

**Conclusions/Significance:**

In this study, we wanted to determine if analyzing completely sequenced cp genomes could help settle these controversies of interspecies relationships in *Camellia* . The results demonstrate that cp genome data are beneficial in resolving species definition because they indicate that organelle-based “barcodes”, can be established for a species and then used to unmask interspecies phylogenetic relationships. It reveals that phylogenomics based on cp genomes is an effective approach for achieving phylogenetic resolution between *Camellia* species.

## Introduction

*Camellia* , a genus containing shrubs and trees, is the largest and most economically, socially, and ecologically valuable genus in the family Theaceae [1–8]. It is native to eastern Asia and is found throughout East and Southeast Asia [3,6], and originated in South and Southwest China [2]. The genus *Camellia* , consisting of more than 200 species [9,10], is not only famous for its ornamental flowers, beverages, and plant oils, but also for its phylogenetic significance. *Camellia* plants provide excellent samples for studying the evolution of the species, interspecific hybridization, and other fundamental life science questions [11]. In addition, *Camellia* tea leaves harbor more than 700 chemical compounds that have been found to promote human health [7,12]. *Camellia* plants are therefore some of the most popular and desirable plants for agriculture, horticulture, and scientific research. Currently, more than 40 countries produce tea for commercial purpose. The annual value of the tea industry in China alone is more than $5 billion USD [12,13]. Many *Camellia* species are domesticated as ornamental plants, while the weeds of others produce high-quality edible oils. Because *Camellia* plants are grown for a variety of uses, they are now found all over the world [3,7,14,15].

Because of their enormous value in commercial, social, and scientific fields, *Camellia* plants have garnered much attention from scientists. The genus *Camellia* represents an excellent example of a taxonomic group under controversial circumscription and having uncertain phylogenetic affinities that require detailed investigations. The traditional classifications of the genus *Camellia* were mainly based on morphology. The three most recently developed traditional classification methods applied to this genus were established by Sealy [16], Chang [9,10,17] and Ming [1,3,5,6], but these systems have given rise to many conflicting results. Sealy, Chang and Ming disagreed on the boundaries of subgenera, sections, and species, as well as the circumscription and relationships between species. Chang identified about 280 species, while Ming only recognized 119 species of *Camellia* . The genus *Camellia* was divided into 12, 20, and 14 sections by Sealy, Chang, and Ming, respectively. Furthermore, the Sealy system did not offer any subgeneric divisions, but Chang divided *Camellia* into four subgenera and Ming divided it into two. So far, it is uncertain which of these systems most accurately describes the phylogenetic relationships within the genus *Camellia* . As a result, it is necessary to seek other evidence that can be used to rebuild the classification system of *Camellia* .

Molecular methods based on DNA sequence analysis provide useful information for taxonomy, species identification, and phylogenetics. In the last few decades molecular phylogenetics has rapidly developed, and is gaining increasing importance in resolving phylogenetic relationships. Efforts to explore the taxonomy issues, relationships, and the evolution of subdivisions in *Camellia* have involved the use of molecular phylogenetic methods [18–27]. Xiao and Parks [22,23] attempted to resolve *Camellia* taxonomy using introns 11-16 and 23 of the RNA polymerase II (RPB2) gene. However, the poorly resolved results of this study presented completely different findings than the traditional classification methods. Another study based on molecular phylogenetics, the Vijayan et al. [7] study, inferred phylogenetic relationships within the genus *Camellia* using internal transcribed spacer (ITS) sequences of 112 species. These results resolved the 112 species into eight major clades, but the interrelationships between clades remained unresolved. Overall, the results from molecular phylogenetic studies have largely differed from the results of studies using traditional classification methods. In addition, recent studies on *Camellia* leaf the morphology have further complicated the classification of *Camellia* [28–32]. Molecular phylogenetic research on *Camellia* has been extensive applied, but there is no apparent structure associated with its molecular phylogeny, which would help to reveal the true phylogenetic relationships between its species. The major reason for the lack of phylogenic structure is because the genus *Camellia* contains a wide variety of species with complex evolutionary relationships. In addition, the lack of appropriate DNA sequences greatly limits the ability to perform adequate molecular phylogenetic research on *Camellia* . Most of the phylogenetic studies performed to date have suggested that the limited availability of suitable DNA sequences has resulted in finding relatively little genetic variation within the genus *Camellia* . Consequently, achieving phylogenetic resolution and performing species identification have been almost impossible. Currently, the interspecies relationships within the genus *Camellia* remain highly controversial.

Owning to the high cost of DNA sequencing and technological restrictions, molecular phylogenetic analyses have typically been limited. These roadblocks severely restricted the extent to which investigators could analyze DNA, only being able to sequence short segments of DNA contain a small number of informative loci. At present, DNA sequencing costs have fallen dramatically with the rapid development of next-generation DNA sequencing technologies [33–38]. Simultaneously, genomics research has also rapid developed. Phylogenomics [39], which combines genomics with phylogenetics, has become an attractive avenue to help reconstruct the tree of life [40]. The technology behind phylogenomics allows large quantities of entire organellar genomes and even nuclear genomes to be rapidly sequenced. Phylogenomics therefore brings the benefits of affordable genome-scale data collection to the area of phylogenetic resolution. As a result, phylogenetic resolution, especially at low taxonomic levels such as genus, has been substantially improved [41].

Plastids are essential organelles in plant cells. Molecular differences that arise in the chloroplast genomes between plant species and individuals offer promising tools to achieve phylogenetic resolution. The chloroplast (cp) genomes in vascular plants have a conserved quadripartite structure composed of two copies of a large inverted repeat (IR) and two sections of unique DNA, which are referred to as the large single-copy (LSC) regions and small single-copy (SSC) regions, respectively [42,43]. There are many advantages to using the chloroplast genome to achieve phylogenetic resolution rather than the nuclear genome, afforded by its haploid nature, maternal inheritance, single structure, gene content, and high conserved genome structure [44,45]. Complete cp genome sequences have been widely used for phylogenetic resolution in plants. Moore et al. [46] resolved the relationships between basal angiosperms using plastid genome-scale data. Similarly, Jansen et al. [47] used 64 plastid genomes to infer relationships between angiosperms. Moore et al. [48] used 83 chloroplast genomes to further resolve the early diversification of eudicots. Parks et al. [41] increased the phylogenetic resolution at low taxonomic levels using chloroplast genomes. Because plastids offer a complete yet relatively small genome, plastid genome sequencing has become a universal method to obtain evolutionary information that can be used for taxonomical and phylogenetic analyses on plants.

Here, we present the complete nucleotide sequences of cp genomes from seven *Camellia* individuals of six species using Illumina sequencing technology applied to total cp DNA. We aimed to evaluate the suitability of using the analyzed cp genome sequences for taxonomy and phylogenetic resolution between *Camellia* species. A phylogenetic tree formed by seven complete cp genomes belonging to six species was reconstructed. Our analyses of seven *Camellia* individuals provided detailed genetic data that was able to differentiate individuals and species. This study supports the method of applying information from complete chloroplast genome sequencing to taxonomy and phylogenetic resolution of *Camellia* .

## Materials and Methods

### Plant Materials

Seven plants from six different species, representing different subdivisions of the genus *Camellia* , were sampled. Healthy, clean, fresh green leaves were collected from the seven adult plants. The voucher herbarium specimens for the seven sampled tea plants were deposited at the Herbarium of Kunming Institute of Botany of the Chinese Academy of Sciences (KUN) (Table S1).

### Chloroplast DNA Extraction, Sequencing, Genome Assembly, and PCR-based Validation

Total DNA enrichment for chloroplast DNA (cp DNA) extraction was performed as described in Zhang et al. [49] from 100 g of fresh leaves. A 5 mg sample of purified DNA was fragmented and used to construct short-insert libraries according to the manufacturer’s manual (Illumina). The DNA from different individuals was indexed using tags and pooled together in one lane of the Illumina’s Genome Analyzer for sequencing at the Beijing Genomics Institute (BGI) in Shenzhen, China. The deep-sequencing datasets of seven plants of *Camellia* were deposited into the NIH Short Read Archive (Table S1).

Because the raw sequence reads included non-cp DNA from the nucleus and mitochondria mixed in with the cp DNA, we isolated the cp sequence reads from the raw sequence reads based on all known angiosperm cp genome sequences. The filtered cp sequence reads were used to assemble cp genomes. First, the filtered short reads were assembled into non-redundant contigs using SOAPdenovo [50], a de novo sequence assembly software, with *k*=31 bp and scaffolding contigs having a minimum size of 100 bp. Then, all contigs were aligned with reference cp genomes, including the cp genomes of plants in the Solanaceae [51,52] and Araliaceae [53,54] families, using the Basic Local Alignment Search Tool (BLAST) database (http://blast.ncbi.nlm.nih.gov/), provided by the National Center for Biotechnology Information (NCBI), using the default search parameters. Next, the order of the aligned contigs was determined according to the reference genomes, and the gaps between the de novo contigs were replaced with consensus sequences of raw reads mapped to the reference genomes. Finally, we acquired preliminary assembly genomes.

The four junctions between the single-copy segments and the inverted repeats were confirmed using PCR-based product sequencing of the preliminary assembled genomes. To avoid assembly errors and to obtain high-quality complete cp genome sequences, we validated genome assembly using intensive PCR-based sequencing. We designed 123 pairs of primers to cover the seven preliminary cp genome assemblies. PCR products were sequenced using the BigDyeV3.1 Terminator Kit for ABI 3730xl (Life Technologies). Sequences obtained using Sanger method were aligned with the assembled genomes using Geneious [55] assembly software to determine if there were any differences. The final complete cp genome sequences of six species of *Camellia* were deposited into the GenBank (Table S1).

### Genome Annotation and Repeat Analysis

We annotated the sequenced genomes using the Dual Organellar GenoMe Annotator (DOGMA) database [56], and then manually corrected for start and stop codons and for intron/exon boundaries in order to match the gene predictions of sequenced cp genomes within GenBank and the Chloroplast Genome Database. The sequences of identified tRNA genes were achieved using DOGMA and tRNAscan-SE (version 1.23) [57]. The functional classification of cp genes was determined by referring referred to the CpBase (http://c hloroplast.ocean.washington.edu/). The annotated GenBank files of the *Camellia* cp genomes were used to obtain gene maps using the OrganellarGenomeDRAW tool (OGDRAW) [58].

Both direct and inverted repeats were assessed using REPuter [59]. Four types of repeats—dispersed, tandem, palindromic, and gene similarity repeats—were determined within the *Camellia* cp genomes. The maximal length of the gap size between palindromic repeats was restricted to 3 kb. Overlapping repeats were incorporated into one repeat motif whenever possible. Furthermore, a given region in the genome was defined as having only one type of repeat, when one repeat motif could be described as both tandem and dispersed, the region was described as a tandem repeat rather than a dispersed repeat.

### Molecular Markers Identification

To examine divergence regions within the seven *Camellia* cp genomes for phylogenetic applications, we extracted all regions, including coding regions, introns and intergenic spacers. Every homologous region was aligned using the Multiple Sequence Alignment Tool (MUSCLE) [60], followed by making additional manual adjustments where necessary. Afterward, the percentage of variable characters within each region was calculated.

For regions that were hotspots of divergence, the maximum parsimony method was used to construct phylogenetic trees using PAUP4.0b10 [61,62], which also allowed us to check the congruence of the phylogenetic tree with the evolution and life history of each species. Heuristic tree searches were conducted using 10,000 random taxon addition replicates (holding 20 trees at each step) and tree bisection-reconnection (TBR) branch swapping with MulTrees in effect. A non-parametric bootstrap analysis was conducted using 1,000 replicates with TBR branch swapping.

### Phylogenomic Analyses

We aligned the seven *Camellia* cp genome sequences using the Muitiple Sequence Alignment Program (MAFFT version 5) [63] and made manual adjustments where necessary. Unambiguously aligned DNA sequences were used for phylogenetic analyses, but ambiguously aligned regions were excluded. To check the utility of different genomic regions for phylogenetic resolution, simultaneous analyses were carried out on the following data: (1) the complete cp DNA sequences; (2) the protein-coding exons; (3) the large single-copy region; (4) the small single-copy region; (5) the inverted repeat region; and (6) the introns and spacers.

Maximum likelihood (ML) and maximum parsimony (MP) analyses were conducted using PAUP 4.0b10. Characters were treated as unordered and unweighted. For ML analyses, the best model and parameter settings were chosen using the Akaike information criterion (AIC) as suggested by Modeltest V 3.7 [64,65]. Heuristic searches were conducted with tree bisection-reconnection (TBR) branch swapping, MulTrees in effect, and 10,000 random taxon addition replicates holding 20 trees at each step. Bootstrap support (BS) values for individual clades were calculated by running 1,000 bootstrap replicates of the data, with starting trees acquired by a single replicate of random stepwise addition of taxa, under TBR branch swapping with MulTrees in effect. The consistency index (CI), retention index (RI) and rescaled consistency index (RC) were obtained through PAUP 4.0b10 as the actual number of site differences excluding insertions and deletions (indels).

Bayesian analyses (BA) were conducted using MrBayes 3.2 software [66,67]. The best model and parameters settings were chosen using the Akaike information criterion (AIC) as suggested by ModelTest v 3.7. The results were based on the best-fit models of the AIC test. Four independent Markov Chain Monte Carlo algorithms were performed simultaneously and sampled every 100 generations for 1,000,000 generations. To establish the burn-in phase, i.e., the phase before the log probability values reached stationarity, we plotted generations against log likelihood scores using Excel 2003 (Microsoft, Redmond, WA, USA); the trees identified in the burn-in period were discarded from the analysis.

## Results

### Genome Assembly and PCR-based Validation

Seven individuals were sequenced to produce 6,539,876 to 7,233,285 paired-end reads (90 bp average read length) using the Illumina Hiseq 2000 system. After screening these paired-end reads by aligning them with reference cp genomes, 108,851 to 112,589 reads were mapped to the reference genomes, reaching, on average, over 100× coverage of the cp genome. After de novo and reference-guided assembly, three complete cp genomes were obtained. The other four cp genomes had four to six gaps, but were complete using PCR-based sequencing.

The four junction regions in each resulting cp genome were validated using PCR-based sequencing. We simultaneously corrected potential errors using PCR-based validation in order to eliminate assembly errors caused by heterogeneous indels from homopolymeric repeats, resulting in complete, high-quality cp genome sequences [38,68]. We designed 123 pairs of primers for the preliminary cp genome assemblies to validate these sequences in each cp genome (Table S2). The validated sequences from each individual reached 172,100 bp. We assembled the high-quality sequences into complete cp genomes using Phred, Phrap, Consed software [69,70]. We then compared these sequences directly to the assembled genomes, and we observed no nucleotide mismatches or indels. These results validated the accuracy of our genome sequencing and assembly methods. We obtained complete cp genome sequences ranging from 156,577 bp to 156,976 bp in length.

### Genome Features and Sequence Divergence

**Figure 1:**
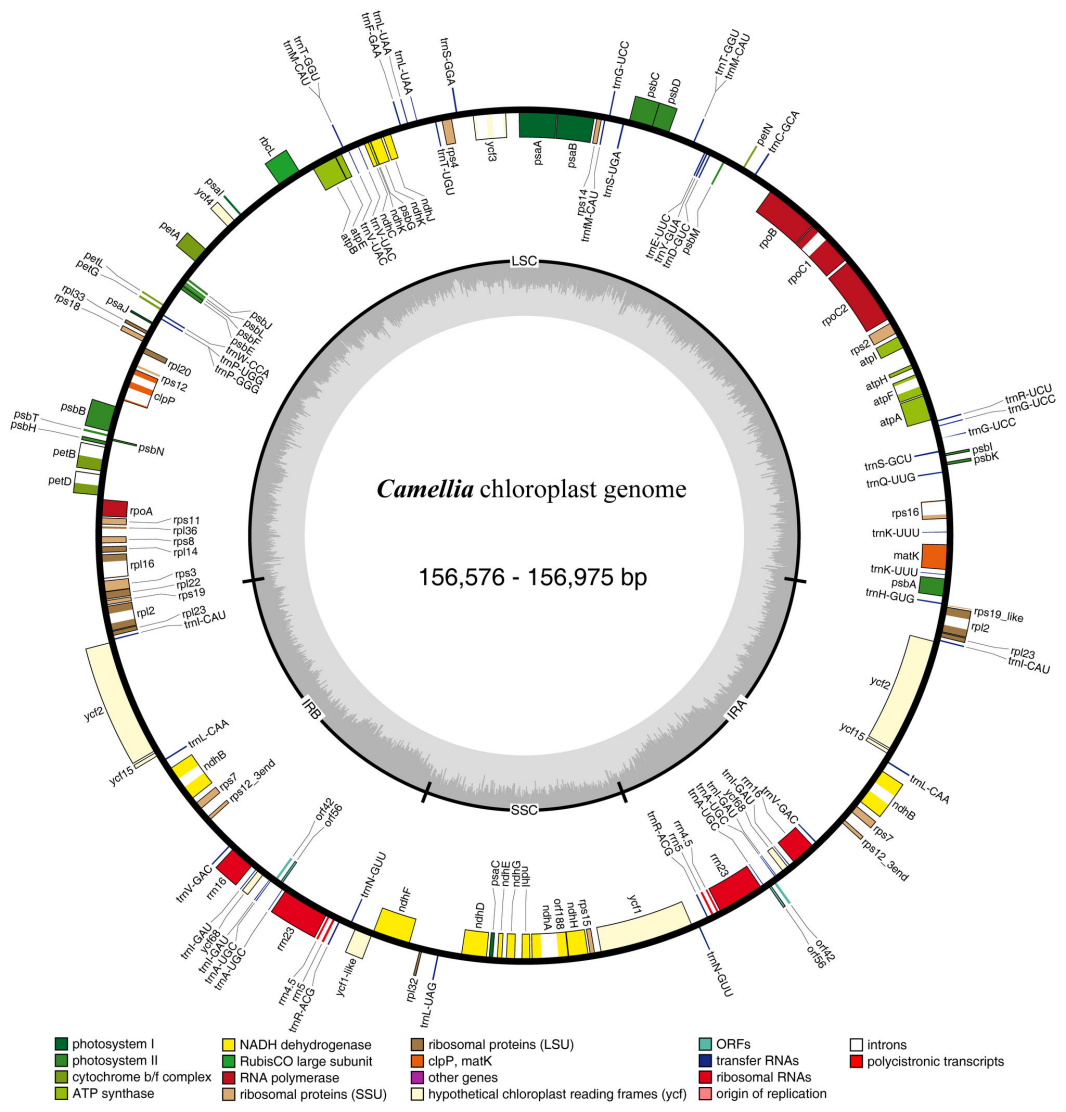
Gene map of the *Camellia* chloroplast genomes. Genes shown outside the outer circle are transcribed clockwise and those inside are transcribed counterclockwise. Genes belonging to different functional groups are color-coded. Dashed area in the inner circle indicates the GC content of the chloroplast genome.

As seen in other angiosperms, *Camellia* cp genomes showed a typical quadripartite structure consisting of a pair of IRs (26,025–26,057 bp) separated by the LSC (86,204–86,673 bp) and SSC (18,232–18,318 bp) regions (Figure 1). The cp genomes were found to encode an identical set of 146 predicted functional genes, of which 123 were unique and 23 were duplicated in the IR regions. The 123 unique genes comprised 81, 38 and 4 protein-coding, transfer RNA and ribosomal RNA genes, respectively. Eighteen distinct genes, namely *atp*F, *ndh*A, *ndh*B, *pet*B, *pet*D, *rpl*16, *rpl*2, *rpo*C1, *rps*12, *rps*16, *trn*A-UGC, *trn*G-GCC, *trn*I-GAU, *trn*K-UUU, *trn*L-UAA, and *trn*V-UAC, contained one intron, while two genes (*clp*P and *ycf*3) contained two introns. These introns of all protein-coding genes shared the same splicing mechanism as group II introns [71]. In addition, we identified some unusual start codons, such as ATC for *ndh*D, GTG for *rps*19. Similar noncanonical start codons have been detected in other angiosperms [68,72] and tree fern plants [73].

We found no genes with lost or reduced functioning in *Camellia* cp genomes. The *ycf*1_like gene in the junction region of IRb and SSC was the only pseudogene found, and arose because of incomplete duplication of the normal copy of *ycf*1 in the IRa and SSC junction region (Figure 1). Similar mutations have been identified in the cp genomes of other angiosperm species [68].

A total of 60.52%-60.71% of the *Camellia* cp genomes were made up of coding regions. Overall, 52.82%-53%, 1.91%-1.92%, and 5.76%-5.78% of the genome sequence encoded proteins, tRNAs, and rRNAs, respectively. The remaining 39.29%-39.48% of the genome was made up of non-coding regions filled with introns, intergenic spacers, and pseudogenes. Similar to other angiosperm cp genomes [72,73], *Camellia* cp genomes was also found to be AT-rich, with overall AT and GC content is 62.7% and 37.3%, respectively. In general, the genome features of the seven *Camellia* cp genomes analyzed in this study were found to be quite similar in terms of gene content, gene order, introns, intergenic spacers, and AT content, and the sequences identity to 98.5%.

**Figure 2:**
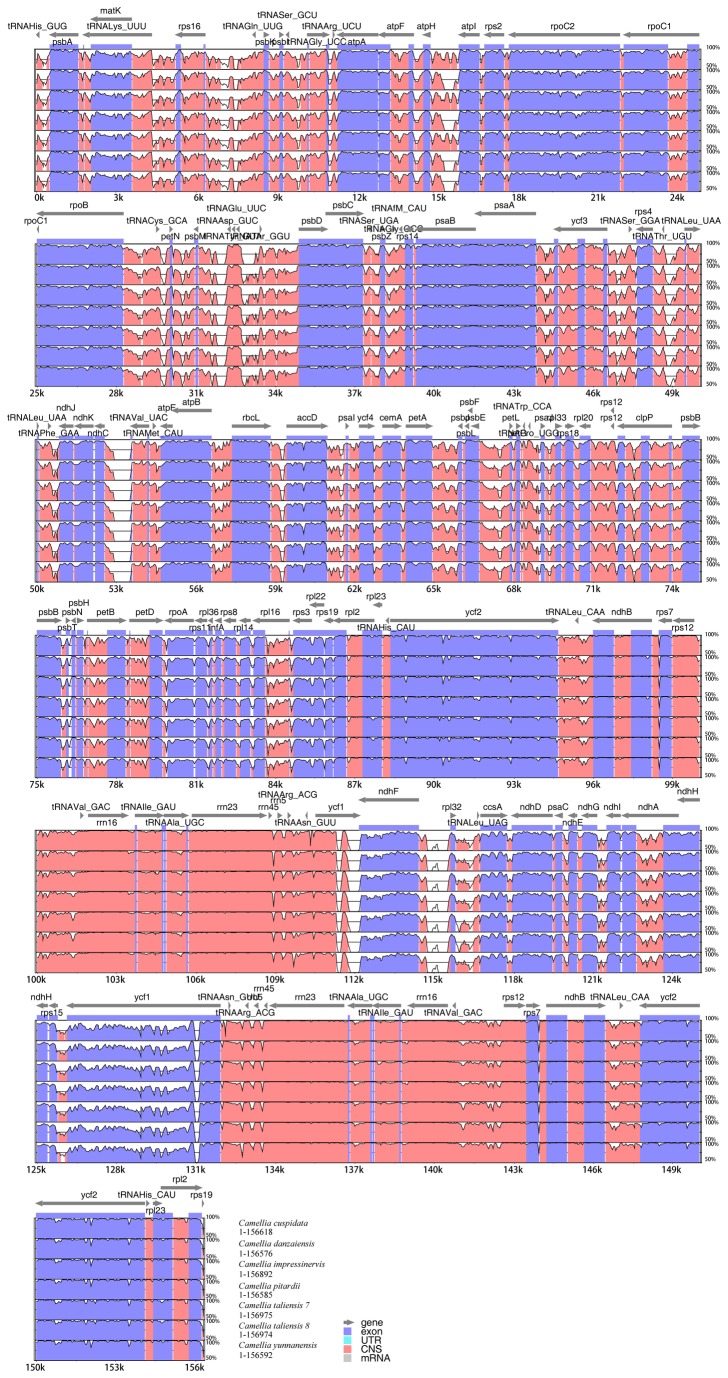
Visualization of alignment of the seven *Camellia* chloroplast genome sequences. VISTA-based identity plots showing sequence identity between seven sequenced chloroplast genomes and the one published chloroplast genomes of Araliaceae, with *Panax ginseng* as a reference. Genome regions are color coded as protein coding, rRNA coding, tRNA coding or conserved noncoding sequences.

Sequences were plotted to check their identity using the mVISTA tool [74] by aligning the seven *Camellia* cp genomes with *Panax* *ginseng* [53] as a reference. The sequences identity percentage is 93% of *Camellia* species and reference. Moderate genetic divergence in *Camellia* species was detected. Taken together, the aligned sequences showed moderate divergence with more than 20 regions having sequence similarities below 60%. These results suggested that *Camellia* cp genomes contain moderate genetic differentiation especially in the noncoding and single-copy regions. More than 10 divergent hotspot regions were identified (Figure 2).

SNPs analyses were conducted using SAMtools [75] and Venn diagram showing overlap of SNPs identified were made (Figure S1). Simultaneously, the variant positions (substitution, indels) and variation types (transition, transversion) were aggregated and summarized according to the coding, intron-spacers, IR, LSC and SSC regions (Figure S2).

P-distances were used to estimate the average genetic divergences of the seven *Camellia* individuals. The results showed that the p-distances in all individuals, between species, and within individuals were 0.000829, 0.00118 and 0.00003, respectively. These results suggest that moderate interspecies genetic divergence existed within the genus *Camellia* . In addition, we found that interspecies sequence divergence was much more pronounced than intraspecies sequence divergence.

### Repetitive Sequences

Four categories of repeats—dispersed, tandem, palindromic and gene similarity repeats [49,76] — were identified using REPuter [59] and manual verification of sequence having a copy size of 30 bp or longer and a sequence similarity greater than 90%. Repeat analysis identified more than 300 repeats in the seven *Camellia* cp genomes. The longest repeat, other than the IRs, was 65 bp in length. Most of the repeated sequences were located in the intergenic regions, while some were found in protein-coding regions.

### Analysis of IRs

Our study showed that the IRs of *Camellia* were representative of the typical dicot cp genome structure, in which the IRs expanded to the *rps*19 and *ycf*1 genes. In IR-LSC, the 5’-end of *rps*19 partially fell within the IRs, and the IRs expanded to the 5’-end of *ycf*1 in IR–SSC.

### Genome Divergent Hotspot Regions

Hotspot regions of sequence divergence were identified using a genome-wide comparative analysis of seven *Camellia* whole cp genomes. The results suggested that 11 hotspot regions (accD *-psa*I, *atp*F*-atp*H, *ccs*A*-ndh*D, *clp*P*-psb*B, *ndh*C*-trn*V, *ndh*F*-rpl*32, *pet*D*-rpo*A, *psb*H*-pet*B, *rpl*32*-trn*L, *trn*G_intron*, trn*S*-trn*G) could be applied to the phylogenetic analysis of *Camellia* . All hotspot regions contained more than 1% variable characters.

### Phylogenomic Analyses

Six data partitions (complete cp DNA sequences, protein-coding exons, the large single-copy region, the small single-copy region, the inverted repeat region and introns and spacers) from the seven *Camellia* cp genomes and four outgroups (NC_006290, NC_016430, NC_004561, NC_ 007062 from GenBank) [51–54] were used for phylogenetic analyses. Excluding outgroups, the sequence characteristics of the ingroups associated with the six datasets are shown in Table S3. The small single-copy region harbored the highest percentage of variable characters, at 0.67%, followed by the introns and spacers with 0.61%. The large single-copy region and the protein-coding exons also possessed moderate genetic variation, reporting 0.48% and 0.34% variable characters, respectively. The inverted repeat region was highly conserved, having the fewest, less than 0.2%, variable characters.

**Figure 3:**
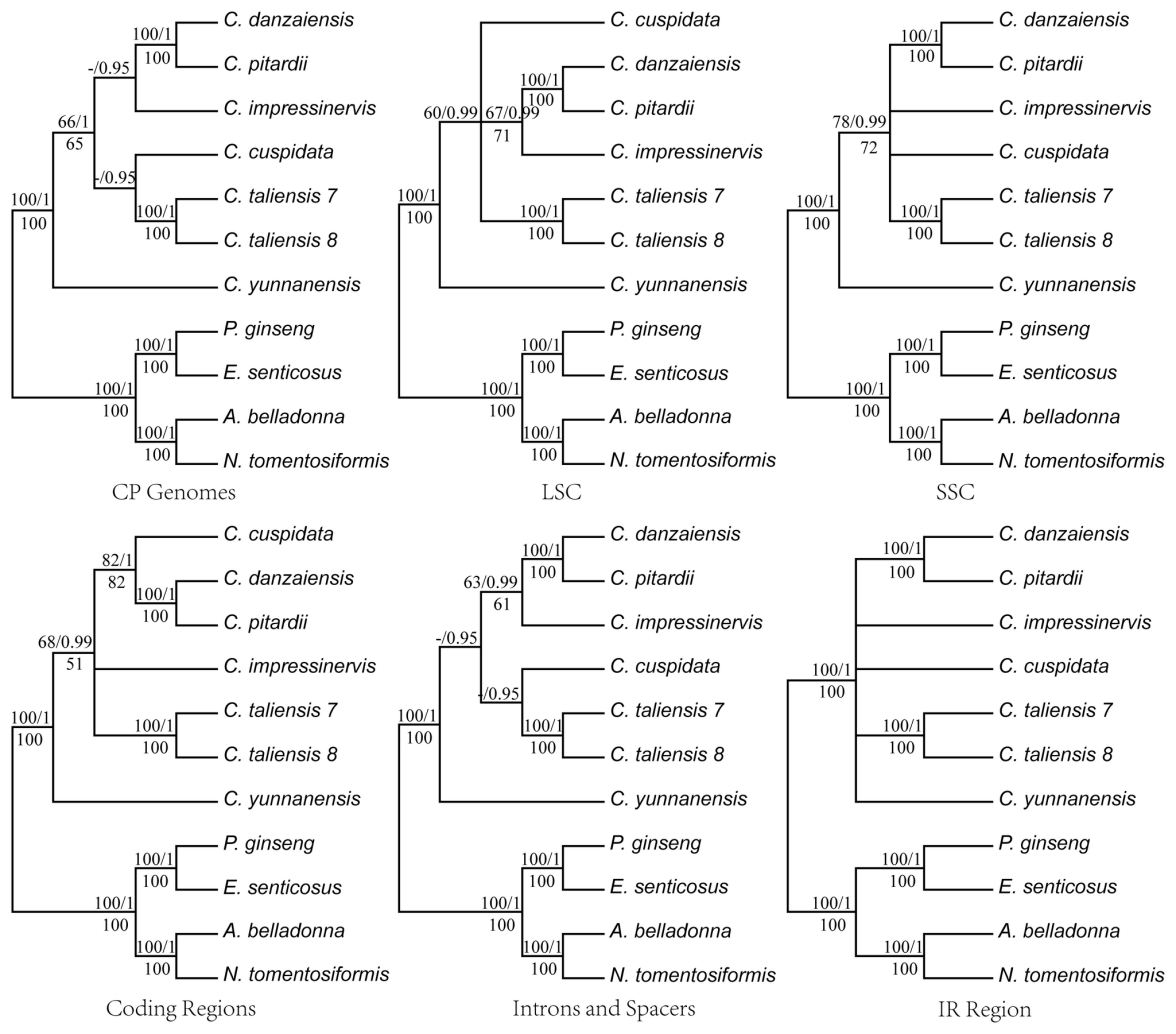
Maximum parsimony trees of all the six chloroplast datasets for seven *Camellia* individuals. Numbers above the lines on the left indicate the maximum parsimony bootstrap of each clade >50%, numbers above the lines on the right indicate the Bayesian posterior probabilities, numbers below each branch are the maximum likelihood bootstrap of each clade >50%.

Phylogenetic trees with bootstrap values (BS) and posterior probabilities (PP) were built based on the previously discussed six datasets (Figure 3). The method of data analysis (ML, MP, or BA) had no effect on the resulting phylogenetic trees, and their topologies were also found to be highly similar. Phylogenetic trees produced according to each of the six datasets were largely congruent with each other. These findings suggest that there were no conflicts between partitions of the cp genome. The results also revealed that the phylogenetic resolution and the support values of nodes increased significantly with the increasing of the sequences (Figure 3).

All analyses (ML and MP) generated a single phylogenetic tree in each dataset. The topology of these phylogenetic trees consisted of dichotomous branches for resolving phylogenetic relationships using the complete cp DNA sequences and the introns and spacers. By contrast, phylogenetic analyses using the other four datasets did not provide much information to help in the phylogenetic resolution of *Camellia* .

## Discussion

### Genome Organization

Structural rearrangements and gene loss-and-gain events often occur in some angiosperms, and are especially common in monocot cp genomes. A representative example is the cp genome of the Poaceae, in which three inversions within the LSC regions and gene translocation of the *rpl*23 gene from the IR to the LSC regions constitutes a disruption to the canonical order [77]. Indels and gene loss (deletions or becoming a pseudogene) are also frequently found in Poaceae cp genomes, as evidenced by intron loss within *rpo*C1, insertion within *rpo*C2, and gene loss in accD, *ycf*1, and *ycf*2 [78,79]. Other monocot families also display rearrangements and gene-loss events in their cp genomes. *Phalaenopsis* and *Oncidium* have lost most of their *ndh* genes [80,81], while *Lemna*, *Dioscorea* and two Acoraceae members each lost a single gene: *inf*A, *rps*16, and accD, respectively [78,82,83]. Rearrangements have also occurred in *Dioscorea* , such as the inversion of SSC [83]. Similarly, rearrangements and gene loss-and-gain events have also occurred in dicots. Geraniaceae cp genomes have experienced remarkable genomic changes [84], such as the loss of *ndh* genes in *Erodium* [85]. Some legumes do not have the IR and have lost the *rps*16 gene [36,86–88]. Usually, IR expansion is quite common, such as the expansion of single-copy *rps*19 and *rpl*22 genes from the LSC into the IRs as a result of gene duplications [89,90]. The *Pelargonium* cp genomes contain massive IR expansions, also due to gene duplications [91]. IR contractions are also common, such as those observed in the subfamily Apioideae [92]. However, we found that the genome organization of *Camellia* wais relatively well conserved. The gene order within the *Camellia* genomes was identical to the gene order in the published Solanaceae and Araliaceae genomes. The cp genomes of the six *Camellia* species were very similar to the cp genome of standard angiosperms, and were distinctly different from the cp genomes of monocots in structure and content. We detected no structural rearrangements, IR expansions, or gene loss-and-gain events in *Camellia* cp genomes. And, as the previous study [93], the *ycf*15 gene, employing an ATG start codon, is likely a functional gene.

### Repetitive Sequences

The presence of repeats in cp genomes, especially in intergenic spacer regions, has been reported in all published angiosperm lineages. Compared with other angiosperm species, the number of repeats found in *Camellia* is rather high. In all, more than 300 repeats were detected in the seven *Camellia* cp genomes. The numbers and distributions of the four repeat types were found to be remarkably similar and conserved among the seven cp genomes. Among these repeats, tandem repeats were the most common, accounting for 42% of the total number of repeats, while gene similarity repeats only made up 4%. Except for a few repeats, which were found in the genes *inf*A, *rpo*C2, *rps*18 and *rps*3, the majority of repeats were located in noncoding regions. The lengths of repeats found in *Camellia* range from 30 to 61, representing much shorter repeats than those in the Poaceae, some of which have measured 91-bp and 132-bp [49,94].

Previous research has suggested that repeat sequences may play roles in rearranging sequences and producing variation which cp genomes through illegitimate recombination and slipped-strand mispairing [76,95,96]. Our research also showed that divergent regions of the cp genome were associated with repeat sequences; for example, the *rpo*C2 gene harbored various repeats. It is possible that repeat sequences also correlate with genome rearrangement in *Camellia* cp genomes.

### Genome Divergent Hotspot Regions

Aligning entire chloroplast genomes revealed that *Camellia* chloroplast genomes are relatively well conserved. Furthermore, similar to other angiosperms, the noncoding regions show greater sequence divergence than the coding regions, among the six *Camellia* species studied. Although the gene order and content between *Camellia* cp genomes were found to be highly conserved, the differences that do exist may indicate of species variation and differentiation. The phylogenetic analyses on the complete cp genomes of six *Camellia* species provided enough evidence for unique variations between the different lineages. The observed rates of interspecies nucleotide polymorphism were moderate at 0.12%.

In this study, 11 hotspot regions of divergence were checked, and were reported to have more than 1% variable characters. Of these regions, 11 intergenic regions harboring high phylogenetic information were newly identified in our study. Previous studies have also shown that noncoding regions of chloroplast genomes could be successfully used for phylogenetic studies in angiosperms [80,97,98]. The new divergence hotspot regions found in our study could potentially be used as molecular targets for future phylogenetic studies. Furthermore, developing universal primers for these hotspot regions could aid in revealing the molecular phylogeny of other *Camellia* species.

### Phylogenetic Implications

Phylogenomic analyses have revealed that different species within a genus are associated with moderate genetic differentiation. Furthermore, individuals of the same species but from different distributions also have moderate genetic differentiation and can therefore be distinctively classified. For example, two individuals of *C* *. taliensis* , both share a common monophyletic node, yet they harbor 16 variable sites. Regardless of the level moderate genetic differentiations may provide enough phylogenetic information to distinguish between species or even individuals. The sites of sequence variation occur primarily in intergenic regions, such as *ndh*C*-trn*V, *pet*D*-rpo*A, *trn*S*-trn*G, etc. The results of our study show that analyses of entire cp genomes significantly contribute to species identification and phylogenetic studies.

Our phylogenetic analysis of *Camellia* did not agree with any of the traditional classification methods used recently in *Camellia* taxonomy. Such as *C* *. danzaiensis* , *C* *. impressinervis* and *C* *. taliensis* , belonging to the Subgen. *Thea* according Chang, did not form a monophyletic clade. Similarly, *C* *. pitardii* and *C* *. yunnanensis* , belonging to Subgen. *Camellia* according to Ming, dispersed into the clade of Subgen. *Thea* comprising *C* *. taliensis* , *C* *. cuspidata* , *C* *. impressinervis* and *C* *. danzaiensis* (Figure 3). Taxonomic studies on *Camellia* are very controversial. Traditional classification systems conflict with each other, especially in terms of species definition; the number of *Camellia* species has been reported any where from 119 to 280, depending on the classification systems used. However, defining species of *Camellia* using analyses of entire cp genomes provides a feasible way to resolve the controversial taxonomy of *Camellia* .

Previous molecular phylogenetic research failed to resolve the phylogenetic relationships of *Camellia* for a variety of reasons. Overall, previous phylogenetic studies did not contain enough informative characters, used samples that may have undergone hybridization, resulted in incomplete lineage sorting, involved stochastic properties, or used non-concerted evolution ITS markers. A comparative analysis using the entire cp genome revealed many informative characters; compared with prior analyses of short sequences in *Camellia* , our analyses on the entire cp genomes contained more than 100 times the number of parsimony-informative characters, and resulted in phylogenetic trees with better-resolved nodes and higher support values. While analyses using entire cp genomes may still be insufficient to fully resolve all phylogenetic relationships [41,99,100], our results suggest that this type of whole-genome phylogenomic analyses will provide solutions to many disputes and guide the way for phylogeny in *Camellia* .

Furthermore, with the rapid development of next-generation DNA sequencing technologies, the sequencing costs have dramatically fallen and the sequencing accuracy has significantly improved. As a result, genome sequencing of organelles and phylogenomic analyses are becoming a reasonable way to improve resolution in phylogenetic studies, especially at low taxonomic levels. In the near future, sequencing the genomes of thousands of organelles will greatly benefit to break the current limitations that arise from using short sequences to carry out phylogenetic studies [41,101,102]. The “barcodes” associated with entirely sequenced cp genomes [101,103] will significantly improve our ability to distinguish between and identify different species. Especially for groups mired in controversy over species definition, organelle-based genome barcodes will help promote taxonomic studies and contribute to the establishment of natural classification systems.

In this study, we sequenced seven individuals, representing six species of *Camellia* using Illumina sequencing-by-synthesis technology. The sequenced cp genomes provided large amounts of genetic information to aid in the species identification and phylogenetics of these economically important plants. The analyzed cp genomes showed moderate genetic variations, which may provide enough genetic information to further species identification and species definition efforts. At the same time, this information may also provide enough adequate phylogenetic information to resolve the evolutionary relationships between species of *Camellia* . Our results show that whole-genome analyses using *Camellia* chloroplast genomes provide an effective and feasible approach to resolve species identification issues and support phylogenetic applications in the study of *Camellia* .

## Supporting Information

Figure S1 Venn diagram showing overlap of seven *Camellia* individuals of SNPs identified. (TIF)

Figure S2 Bar graph summarizing the variant positions and variation types in the different regions. (TIF)

Table S1 Sampled species and voucher specimens of *Camellia* used in this study. (DOC)

Table S2 Primers used for gap closure, assembly and junction verification. (DOC)

Table S3 DNA site variation and tree statistics for the six datasets used in the phylogenomic analyses presented in this study. (DOC)
