# Supplementary figures and images for: Comparative Chloroplast Genomes of Camellia Species
Source: PLoS One. 2013 Aug 23;8(8):e73053. doi: 10.1371/journal.pone.0073053 (PMC3751842; doi:10.1371/journal.pone.0073053)

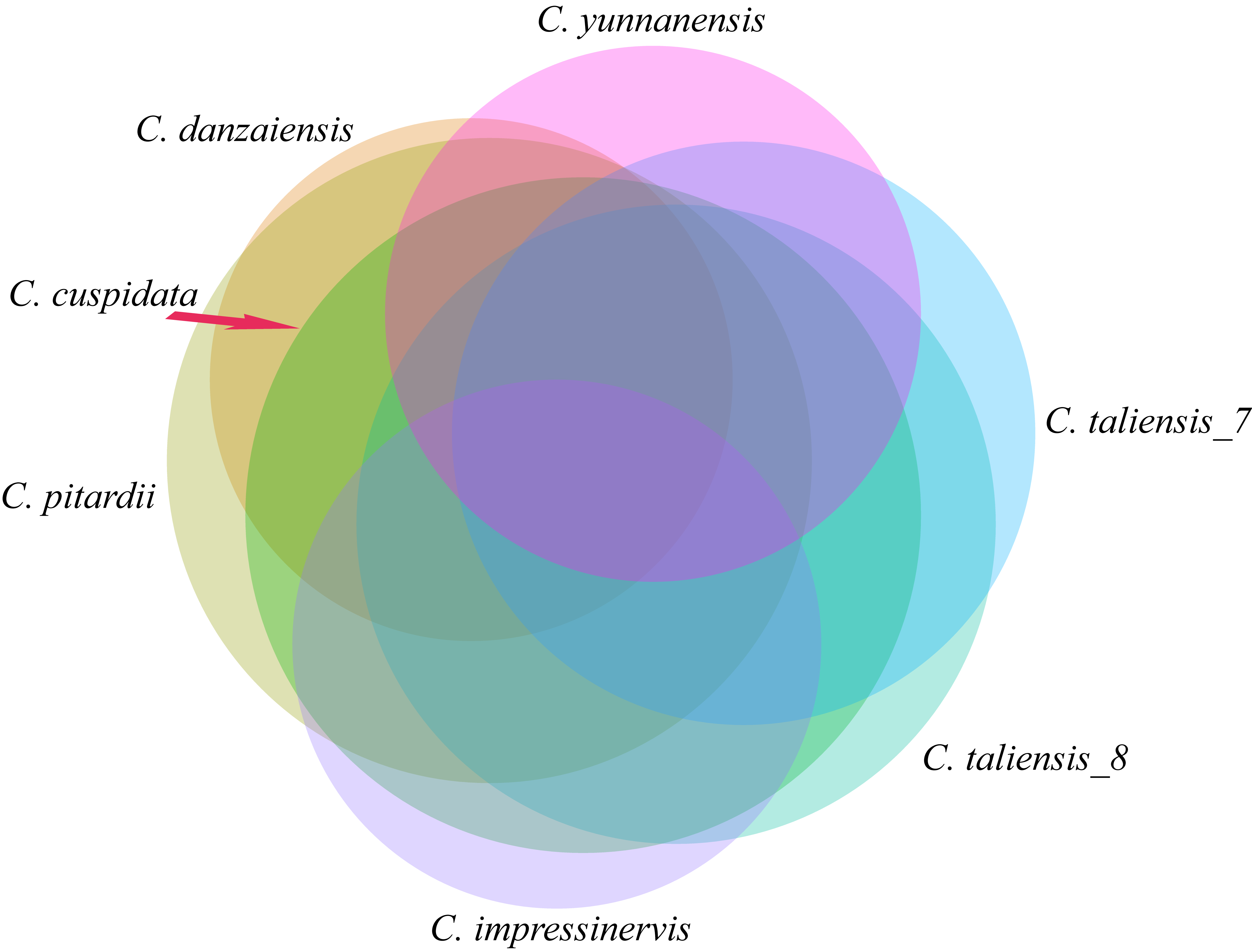

Supplement: Figure S1 — (TIF) [file pone.0073053.s001.tif]

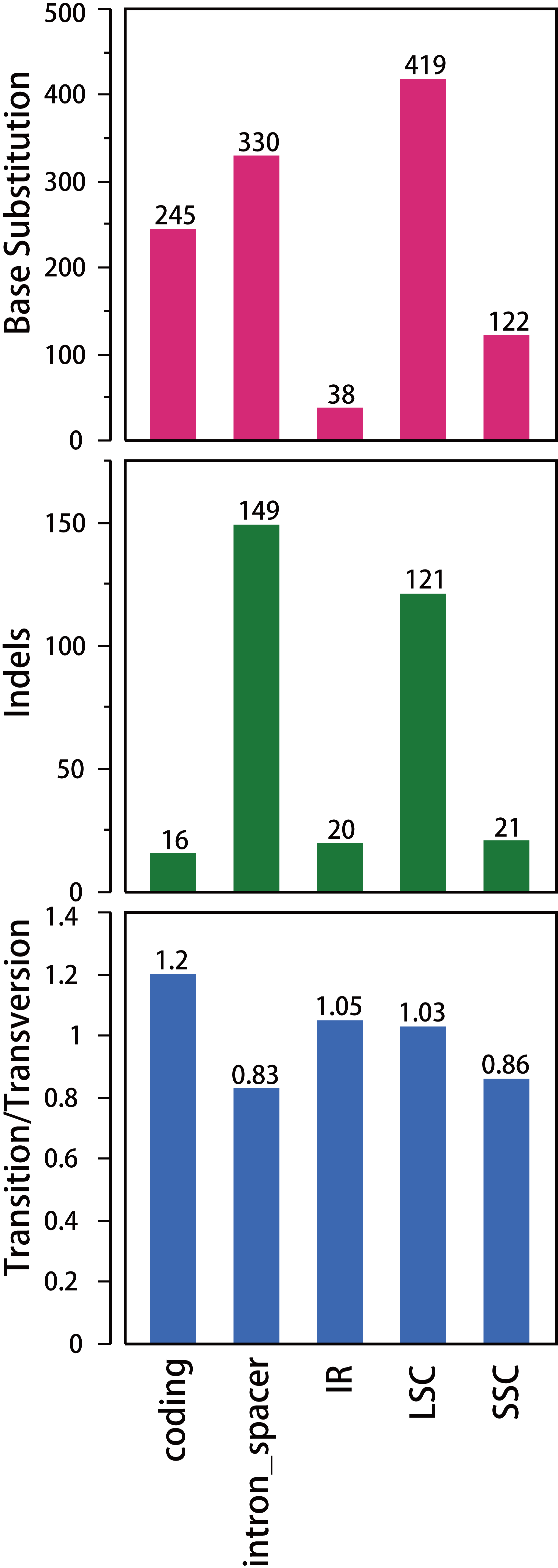

Supplement: Figure S2 — (TIF) [file pone.0073053.s002.tif]
