# Supplementary material for: Comparative Chloroplast Genomes of Camellia Species
Source: PLoS One. 2013 Aug 23;8(8):e73053. doi: 10.1371/journal.pone.0073053 (PMC3751842; doi:10.1371/journal.pone.0073053)
Supplement: Table S1 — (DOC) [file pone.0073053.s003.doc]

Table S1. Sampled species and voucher specimens of *Camellia* used in this study.

| Taxon | Geographic origin | Voucher | GenBank accession | NIH Short Read Archive accession |
| --- | --- | --- | --- | --- |
| *Camellia impressinervis* | KIB | S.X.Yang1080 | KF156835 | SAMN02183179 |
| *Camellia danzaiensis* | Danzhai, Guizhou | S.X.Yang3147 | KF156834 | SAMN02183178 |
| *Camellia pitardii* | Danzhai, Guizhou | S.X.Yang3148 | KF156837 | SAMN02183180 |
| *Camellia cuspidata* | Zhenyuan, Yunnan | S.X.Yang3159 | KF156833 | SAMN02183177 |
| *Camellia taliensis_7* | Zhenyuan, Yunnan | S.X.Yang3158 | KF156836 | SAMN02183176 |
| *Camellia taliensis_8* | Zhenyuan, Yunnan | S.X.Yang3157 | KF156839 | SAMN02183175 |
| *Camellia yunnanensis* | KIB | S.X.Yang1090 | KF156838 | SAMN02183181 |

KIB: Kunming Botanical Garden of the Kunming Institute of Botany
