# Supplementary material for: Comparative Chloroplast Genomes of Camellia Species
Source: PLoS One. 2013 Aug 23;8(8):e73053. doi: 10.1371/journal.pone.0073053 (PMC3751842; doi:10.1371/journal.pone.0073053)
Supplement: Table S2 — (DOC) [file pone.0073053.s004.doc]

Table S2. Primers used for gap closure, assembly and junction verification.

| Primer | Forward sequence | Reverse sequence |
| --- | --- | --- |
| P1 | 5' AAACCCTTTTGTAGCGAATCCGT 3' | 5' TGGTGTATTCGGCGGCTCCCT 3' |
| P2 | 5' AGGATGTTGTGCTCAGCCTGGA 3' | 5' GGTTCGAATCCCGGGCAACCC 3' |
| P3 | 5' ACAATGGGTTGCCCGGGATTCG 3' | 5' TGGGCTATCTTTCAAGTGTGCGAC 3' |
| P4 | 5' CCTTGAACAACCACAGAATGACCTGA 3' | 5' TCAGCAACACGACTTCGTATATCCAC 3' |
| P5 | 5' AGTGCGATACGGTCAAAACAAGGT 3' | 5' ACCACGACTGATCCTGAAGGGGA 3' |
| P6 | 5' AGGGTGTGTAGATACAATCAGAATCA 3' | 5' TCTCGAGCCGTACGAGGAGA 3' |
| P7 | 5' TCTCCTCGTACGGCTCGAGAAA 3' | 5' TCGCGTGGGAATCAACCATTTGT 3' |
| P8 | 5' ACTCGAGCTTCATCGTGGACT 3' | 5' TCGCTCTATTTCAGTGACAGTAGTT 3' |
| P9 | 5' ACTGTCACTGAAATAGAGCGATAACA 3' | 5' TCGAAATGCGAAAGAACCTATATTTCC 3' |
| P10 | 5' ACATATGATGAAAAGCCCGCCCAA 3' | 5' CGGCAGCTTGCCAAACAAAGGC 3' |
| P11 | 5' CCTTTGTTTGGCAAGCTGCCGT 3' | 5' GACCTAGCCCGGCCATGGGA 3' |
| P12 | 5' TCCCATGGCCGGGCTAGGTC 3' | 5' TCACGAGAATGCATATTTTTCCTCGAA 3' |
| P13 | 5' TCCATGGATGAAGATAGAAAAGTAGA 3' | 5' TCACCGAGGAAGTGGAAGCCCT 3' |
| P14 | 5' AGATAGGATTTCTTGGAACTGAGGT 3' | 5' ACAAGCACAAGCTTATCGCCA 3' |
| P15 | 5' TGGCGATAAGCTTGTGCTTGTTTGG 3' | 5' TGGTATGTTTGGGGCGATGAAAGAAA 3' |
| P16 | 5' TCCATGATCCCTTCCCGAACCA 3' | 5' TGTCCTTCCCCTATTCGTCCAAGGG 3' |
| P17 | 5' AGTGCTAATGCAACAACCAGTCC 3' | 5' AGTCAACTCTTGTAGTGTGGGTGT 3' |
| P18 | 5' CCCAGTTCGGCTCTCCCTCTCC 3' | 5' TCACTTAGTTTTCGACTTTTCGGGAAT 3' |
| P19 | 5' ACCCAAGGACCATATTCTTCCCCAA 3' | 5' GCACGGGCTGCAATAAGGGCT 3' |
| P20 | 5' ACGCTTTGCAGAGATATAAGGTGCCA 3' | 5' CCAAAAGGTTTATCGATCCCAAGGGG 3' |
| P21 | 5' TGGATCTGTACCCCTTGGGATCG 3' | 5' AGGATCCGTCGAGTTCAGGATTGA 3' |
| P22 | 5' AGGAAGCACGGGCCTCTTCG 3' | 5' AGGCGGGTTAGTCCGAGTGGA 3' |
| P23 | 5' AATTTGTGCCTCACTTGATCATTAGTC 3' | 5' TGTCGGATCCCCAAGGACAA 3' |
| P24 | 5' TGGATGTAGCAGTGGCTTGCTGG 3' | 5' TCTTGCTTCGAACATAGGAGTTGCG 3' |
| P25 | 5' CCTGGCACATTACTAATTCTCCTGGCG 3' | 5' TGGTGTATCTGCTTGAGCCGGA 3' |
| P26 | 5' AGGTCCCAACTTCACCTCGAC 3' | 5' AGGAAGGAAGCTTAATCGGAATGAATC 3' |
| P27 | 5' TGATTCATTCCGATTAAGCTTCCTTCC 3' | 5' ATGAAGCCAGTAAGCAAACAGCGA 3' |
| P28 | 5' TGTCTTCCAGCTACTTTATCGCCT 3' | 5' TCCATTAGTTATGTATCAGCGTTCCA 3' |
| P29 | 5' TCGAAGATGAACCTGTTCCCATGC 3' | 5' TGTGTAGGCGGGAATCCGGT 3' |
| P30 | 5' AAAATTTCAGGATAGCAAACATTCTCT 3' | 5' TGCTACGGGATGGAAATGAAGGA 3' |
| P31 | 5' GCCACTTAGACTTATTATGTTATGGGA 3' | 5' TCCCCCGCCTTACCACTCGG 3' |
| P32 | 5' CCCGGTTCAAATCTGGGTGTCGC 3' | 5' CTTGACTAGGGGTTTGCCGAAAAGA 3' |
| P33 | 5' GTTTTGCAAAGCGTTTTTAACGA 3' | 5' TCGGCAATATTCTATGCGATCCGA 3' |
| P34 | 5' ACTATCGGATCGCATAGAATATTGCCG 3' | 5' TGGTCAGAGCACCGCCCTGT 3' |
| P35 | 5' ACTACAATCCCAGGGAAATAAGGGA 3' | 5' AGTGGCTAATTAGTAGCTCATTCTTGA 3' |
| P36 | 5' TGGTAGAGTAACGCCATGGTAAGGC 3' | 5' TCGCTACCAGTTGATCCTCAGACTT 3' |
| P37 | 5' ATGGAACAAGGGAATAAGTCTGAGGA 3' | 5' GCCTACAAAAACGAAACGGTCCCT 3' |
| P38 | 5' GGGGGTAGGTCCTGGTGGGG 3' | 5' CCCTTCCCGCGTGCCACAAA 3' |
| P39 | 5' ACCTGTTCTTTCCATGACCCCTCT 3' | 5' GGGGTCATTTCGTTTTTGGAGCTGG 3' |
| P40 | 5' AGCTCCAAAAACGAAATGACCCCC 3' | 5' TGGCAAGGAAAAGTTTGATTCAGAGGG 3' |
| P41 | 5' ACCCGATGTAGAGGCAATCAAGAAAGC 3' | 5' ACGAGTGGCCCGGCATTCAA 3' |
| P42 | 5' GGGCCAAGAGCACCACCTCG 3' | 5' TCATGCATTTACGATTCATGTGACGGT 3' |
| P43 | 5' ACGAGCAAATAGAACACCTTTCAGGAG 3' | 5' GCTACTGACTACGGTACACAACTGTCA 3' |
| P44 | 5' AAATGGGGTTGCCCCCTCGG 3' | 5' TGTGTTGTCCGGAAAGAGGAGGA 3' |
| P45 | 5' GCCATGAGGCGCTCAACGGA 3' | 5' TGCTTTATTCTTCTGAAGGTGGGAGA 3' |
| P46 | 5' TCTCCCACCTTCAGAAGAATAAAGCA 3' | 5' ACCATCGAAGGCTGTTGAAGTGAC 3' |
| P47 | 5' GTGTCTTTCTCGTAAGACTGAGAGCAG 3' | 5' ACCCAAAAACCGAGTGAATAGCGGG 3' |
| P48 | 5' TGGCCGAGTGGTTCAAGGCG 3' | 5' ACCAAGTTATCGTTGCAAACCCCA 3' |
| P49 | 5' TCCCGGAACGCGATTGGTTT 3' | 5' TGGAATACTTGAACGGTCGATTCTTTT 3' |
| P50 | 5' GGCAAGAAATCAAAGAAGAGAAAACAC 3' | 5' CAGGCCCTGGAATTTCTTGGA 3' |
| P51 | 5' CGGGATAGCTCAGCTGGTAGAGCA 3' | 5' TCATCCGCGCTTGAAACGTATCTT 3' |
| P53 | 5' CGAGGGATCTCTGAAGTAGATGGTGGT 3' | 5' CCAATGGGCGACGCTTGGTT 3' |
| P54 | 5' CGTAAAGCAGAAACATAGACGCACTCC 3' | 5' ACCAAACGTGACCTATCGCATCG 3' |
| P55 | 5' TCGATGCGATAGGTCACGTTTGGT 3' | 5' TCGATGAAGCTACCGCGAAGGC 3' |
| P56 | 5' ACGCTCAATTTTTCGTGCTCTTGC 3' | 5' GCCCCTTATCGCCGTGGAGG 3' |
| P57 | 5' TCTTGCCCGGCGGAAAGGAT 3' | 5' GCGGCCCCTGCTTCTTCAGG 3' |
| P58 | 5' TGCTTATGTAGCGTATCCTTTAGACCT 3' | 5' CCGGTGCATTTCCCCAAGGGT 3' |
| P59 | 5' AGCTGCTGCTTGTGAGGTATGGA 3' | 5' TCTATTCGGATCTTCACTTTCACTGGT 3' |
| P60 | 5' TGATCATTTATTCGGCGTCAAGGACA 3' | 5' CGTGCACCCCCAGAAGCACA 3' |
| P61 | 5' TCCTACTACTGGTGGGGTGACAGC 3' | 5' TGTTCGGCAGAAGCCGCATGT 3' |
| P62 | 5' TCTAGCTATTTTCAAATCCAATCGGGA 3' | 5' CAAGGGAATGGCCCCCTGGC 3' |
| P63 | 5' AGAATGAATGCTTTCTCAGCAGGAG 3' | 5' TTTGTGATTCCGTGGTTAGTACTTGAA 3' |
| P64 | 5' AGTCGGCGAATGAGGTGGGT 3' | 5' TGCATGGTCCAATCATTGATCCCG 3' |
| P65 | 5' GCCCCTCCTGATCGTATTTCTCCCG 3' | 5' ACCAGGAATGTGTGCGGTACAAGG 3' |
| P66 | 5' TGTACCGCACACATTCCTGGTCA 3' | 5' GGGAAGGCCCCGTTGAGTTCT 3' |
| P67 | 5' ACCGATCACAAGAATACCAGTTACAGT 3' | 5' TGAAATTGCGTTGCTGTGTCAGAA 3' |
| P68 | 5' TCCCACAATTTAAGTAGATGCGAGAT 3' | 5' CAAATAAGTCGTATCTTGCTCAGACC 3' |
| P69 | 5' AGCGGCTTTAATTATAACCTCAGCTC 3' | 5' AGGGACACTTGTAGTTGTACACAGA 3' |
| P70 | 5' TCCGGATGCGTTAACATTCCCCTT 3' | 5' CCTTGGAAGGGTAACACACAGGTGC 3' |
| P71 | 5' CCGAACCGAGCACCTGTGTGT 3' | 5' CGTGGTCGGGAAGGTTATAGTAGCCA 3' |
| P72 | 5' TGGCTACTATAACCTTCCCGACCACG 3' | 5' TGTCAGCAACAGAAGCCCAAGC 3' |
| P73 | 5' AGCTTGGGCTTCTGTTGCTGACA 3' | 5' TGCATGGGATTAGCTGCTTCAATGGG 3' |
| P74 | 5' GGCTACTCCAGGTATTACCCATCCACC 3' | 5' GCAAAAAGAAGTGGTAAGGGGAGCA 3' |
| P75 | 5' ACGAAAAGGCACTTTTGGAACACCA 3' | 5' ACCAGCCCAACCAGCAACCA 3' |
| P76 | 5' TCAGGGGTGGCTTGCTTTGGT 3' | 5' TCCATTGAGTTCGCCGCCGT 3' |
| P77 | 5' CGGGCACATTTGGCATGGTGC 3' | 5' ACCGCTTTTGGGCAACCTTCTCA 3' |
| P78 | 5' AGAACCGCAAAATCCTAGCTTTCA 3' | 5' TCGCCTGAATTTCGAGACGTTCTT 3' |
| P79 | 5' TCGTCCGACCGTTACTGAGGCT 3' | 5' ACGGCGGTAAGAAGAGGCAATACA 3' |
| P80 | 5' TGGATTATGGGAGTGGGTGACTTGA 3' | 5' GCCATGCGGGCTCTCCGTAA 3' |
| P81 | 5' TGGTGAACCGGCAGATCCAT 3' | 5' CATGAAGCTTCTCGGAATTTGATTGA 3' |
| P82 | 5' AGCTTCATGAAATGCTTCTTTAGGAGT 3' | 5' CTGCTCAAACCGCAGCGGGA 3' |
| P83 | 5' ACGTGAACCAATACGTCCATTCCTACG 3' | 5' TCGAGAACGTGAGGAAACAACAAGAA 3' |
| P84 | 5' TGTCAGCAACAGTGTCCCTACCCA 3' | 5' ACACGTATGGGTTCGGGGAAAGG 3' |
| P85 | 5' AGGAGATCCTTTCCCCGAACCCA 3' | 5' AGCCTCGAGCCAATAAAGACTGAGA 3' |
| P86 | 5' TGCTTTATTACATTGCCTTTTTGAGA 3' | 5' TGCAAAACCTTATGGAAACCCT 3' |
| P87 | 5' GGGTTTCCATAAGGTTTTGCAATTCT 3' | 5' TGGTAGGTCACAAGTTGGGAGA 3' |
| P88 | 5' ACGGTAGTACCCTCGTTGACTTCG3' | 5'GCAACAGTCGGACAAGTGGGGA 3' |
| P89 | 5' TCCATTGCGTGTGCTCGGGT 3' | 5' TGGGGCATACAATGCATTACAGACG 3' |
| P90 | 5' TGAAACCGTCGCTGGAATTGAGA 3' | 5' TGGTTAGAATCCGTTACTTGAACGAAA 3' |
| P91 | 5' AGCACCTATGGGTACATAAGAAATGT 3' | 5' TGCAGAGTTGATCATTCGGACCT 3' |
| P92 | 5' AACAACCGGGAGCAATTTACTTACGA 3' | 5' GCAGCTCCGTATCAAGGTCACGA 3' |
| P93 | 5' AGATGGCTTTTCCCGGATGAAATGAA 3' | 5' AGAGCGTGGAGGTTCGAGTCC 3' |
| P94 | 5' TCGACCTTGGTTCCGTAGGAGCA 3' | 5' GCCTTTCATTTGCTTCTCTTCGATGG 3' |
| P95 | 5' TCGTTTCCTTTTCGTTTGTTTCGAG 3' | 5' TCGGAAACCCAAGGACTCAATCGT 3' |
| P96 | 5' CTCCTCCTTTCTTCCACTCCGTCCC 3' | 5' CTGTCTTTTCTGTATCCTTTCCCCGGT 3' |
| P97 | 5' TGTAGTGACGCATCTTGTATGTGTTCC 3' | 5' TGGGTTTAGGGATAATCAGGCTCGAA 3' |
| P98 | 5' TTCCAGTGGCGGACGGGTGA 3' | 5' TTCCACCGCCTGTCCAGGGT 3' |
| P99 | 5' AGGCTGCAACTCGCCTGCAT 3' | 5' CACATCTCAGGGTCAGGCGCT 3' |
| P100 | 5' GGTTTAGGTTCGGCCTCAATGGGA 3' | 5' GCCGCCGACTCCAACTACCG 3' |
| P101 | 5' GTCGGGGGACCTGAGAGGCG 3' | 5' AGCTCTGGGTTCGAGTGGCA 3' |
| P102 | 5' GTCCCGAGGGACGGAGGAGG 3' | 5' ACAGCCCCAGGTGGCGAAGA 3' |
| P103 | 5' TCGATCAGGCACTCGCCATCT 3' | 5' AGGCCACAATTACCGCGAGCA 3' |
| P104 | 5' TCCCGTTCTCAACCCATGACCA 3' | 5' AGAATGGGTGACGGTATTCTGCCT 3' |
| P105 | 5' CGGAAGAAAAGGAGGATCCGGACA 3' | 5' TTTCAATCATGAAAGAATGGCTTTGGA 3' |
| P106 | 5' CCAACAAACAAAGTAAATAGGACCA 3' | 5' TGCCATGGAAGGGCCTACTCCT 3' |
| P107 | 5' GCCCTTGGAAAAAGAAGAAGTCCCGC 3' | 5' CCGGTTGAAAGAGATTTCCCTAACG 3' |
| P108 | 5' TCGTTAGGGAAATCTCTTTCAACCGGA 3' | 5' TCCTAAGAGCAGCGTGTCTACCA 3' |
| P109 | 5' TTCAATTCCCGATTTCCATTCCGTCA 3' | 5' TGACCCCCATGCTTCATTAGCCCA 3' |
| P110 | 5' AGCCGGCGAGAACCATGTGA 3' | 5' GGGAGCGTATGGGTTGGTTCGG 3' |
| P111 | 5' AATCGGTGATTTGACAGCAAAAGCAAT 3' | 5' TGTGTCCGAGCCTGTCCCACA 3' |
| P112 | 5' TGTGGGACAGGCTCGGACACA 3' | 5' AGGTGCAATCGCTGTAGCTCGT 3' |
| P113 | 5' TCCATCTCCAACGGTCCAAAGA 3' | 5' TGATTGGTTGGTGAAAATTACCATTGA 3' |
| P114 | 5' GCCCCGGAATCGCTCCGATG 3' | 5' TGTGACCCTCGAGGAGCCGT 3' |
| P115 | 5' ACGGCTCCTCGAGGGTCACA 3' | 5' TGAAAGAAAACGGCTTATAAATTCGCA 3' |
| P116 | 5' AAGCCGTTTTCTTTCACTCATAGAACT 3' | 5' GCTCTGGAAGGAATTCCGGGGG 3' |
| P117 | 5' CCCCTTCTTTTTGCCATTGGACTTCCC 3' | 5' AACGTCAACGCCTGCTCGCT 3' |
| P118 | 5' AGCGAGCAGGCGTTGACGTT 3' | 5' TGGCAGTCGTTTCAGCATGTTAAGG 3' |
| P119 | 5' TCGACTCCAATTTTTGACCTTGTTCCC 3' | 5' TTACGATTCCTCAGGATCAAGAAATCA 3' |
| P120 | 5' TGATTTCTTGATCCTGAGGAATCGT 3' | 5' TCAATGGAAAAACTGGTTAAGAGGTCA 3' |
| P121 | 5' ATGACCTCTTAACCAGTTTTTCCATTG 3' | 5' CCTTTCTGTCTGAAACCCTGGCACA 3' |
| P122 | 5' TCCTCAGACCACTCAGGAAATTGT 3' | 5' AGGATTAGATTGTGTAATGATGAGCCT 3' |
| P123 | 5' TGTTTGTTGGTCGAAACCCAATGGT 3' | 5' TGGATTGGTATTAGTCTGGATACGGCA 3' |

Primer pairs P1, P88, P105 and P123 were used to verify Joint IRa/LSC, LSC/IRb, IRb/SSC and SSC/IRa, respectively.
