# Supplementary material for: Comparative Chloroplast Genomes of Camellia Species
Source: PLoS One. 2013 Aug 23;8(8):e73053. doi: 10.1371/journal.pone.0073053 (PMC3751842; doi:10.1371/journal.pone.0073053)
Supplement: Table S3 — (DOC) [file pone.0073053.s005.doc]

Table S3. DNA site variation and tree statistics for the six datasets used in the phylogenomic analyses presented in this study.

|  | Variable sites (ingroups ) (%) | Informative sites (ingroups ) (%) | Trees (n) | Tree length | CI | RI | RC |
| --- | --- | --- | --- | --- | --- | --- | --- |
| complete cp genomes | 0.39 | 0.17 | 1 | 21910 | 0.976 | 0.979 | 0.956 |
| coding exons | 0.34 | 0.16 | 1 | 8887 | 0.972 | 0.976 | 0.949 |
| LSC region | 0.48 | 0.21 | 1 | 16234 | 0.978 | 0.981 | 0.960 |
| SSC region | 0.67 | 0.29 | 2 | 4708 | 0.970 | 0.974 | 0.945 |
| IR region | 0.15 | 0.08 | 2 | 955 | 0.981 | 0.985 | 0.967 |
| introns and spaces | 0.61 | 0.25 | 1 | 12513 | 0.981 | 0.982 | 0.964 |

CI, consistency index; HI, homoplasy index; RI, retention index; RC, rescaled consistency index.
